# Supplementary material for: HIVprotI: an integrated web based platform for prediction and design of HIV proteins inhibitors
Source: J Cheminform. 2018 Mar 9;10:12. doi: 10.1186/s13321-018-0266-y (PMC5845081; doi:10.1186/s13321-018-0266-y)
Supplement: Supplementary file 2 — Additional file 2. Source code of HIVProtI web server. [file 13321_2018_266_MOESM2_ESM.zip › HIVprotI_Source-code/hivprotI/weka-3-6-6/doc/weka/filters/unsupervised/attribute/package-tree.html]

weka.filters.unsupervised.attribute Class Hierarchy


---


|  |  |  |
| --- | --- | --- |
| **Overview** \| **Package** \| Class \| **Tree** \| **Deprecated** \| **Index** \| **Help** \| **Weka's home** | |  |
| **PREV**   **NEXT** | **FRAMES**    **NO FRAMES**     **All Classes** |  |


---


## Hierarchy For Package weka.filters.unsupervised.attribute

**Package Hierarchies:**: All Packages

---

## Class Hierarchy

- java.lang.Object
  - weka.filters.**Filter** (implements weka.core.CapabilitiesHandler, weka.core.RevisionHandler, java.io.Serializable)
    - weka.filters.unsupervised.attribute.**AbstractTimeSeries** (implements weka.core.OptionHandler, weka.filters.UnsupervisedFilter)
      - weka.filters.unsupervised.attribute.**TimeSeriesTranslate**
        - weka.filters.unsupervised.attribute.**TimeSeriesDelta**
      - weka.filters.unsupervised.attribute.**Add** (implements weka.core.OptionHandler, weka.filters.StreamableFilter, weka.filters.UnsupervisedFilter)- weka.filters.unsupervised.attribute.**AddCluster** (implements weka.core.OptionHandler, weka.filters.UnsupervisedFilter)- weka.filters.unsupervised.attribute.**AddExpression** (implements weka.core.OptionHandler, weka.filters.StreamableFilter, weka.filters.UnsupervisedFilter)- weka.filters.unsupervised.attribute.**AddID** (implements weka.core.OptionHandler, weka.filters.UnsupervisedFilter)- weka.filters.unsupervised.attribute.**AddNoise** (implements weka.core.OptionHandler, weka.filters.UnsupervisedFilter)- weka.filters.unsupervised.attribute.**AddValues** (implements weka.core.OptionHandler, weka.filters.StreamableFilter, weka.filters.UnsupervisedFilter)- weka.filters.unsupervised.attribute.**ChangeDateFormat** (implements weka.core.OptionHandler, weka.filters.StreamableFilter, weka.filters.UnsupervisedFilter)- weka.filters.unsupervised.attribute.**ClusterMembership** (implements weka.core.OptionHandler, weka.filters.UnsupervisedFilter)- weka.filters.unsupervised.attribute.**Copy** (implements weka.core.OptionHandler, weka.filters.StreamableFilter, weka.filters.UnsupervisedFilter)- weka.filters.unsupervised.attribute.**FirstOrder** (implements weka.core.OptionHandler, weka.filters.StreamableFilter, weka.filters.UnsupervisedFilter)- weka.filters.unsupervised.attribute.**MakeIndicator** (implements weka.core.OptionHandler, weka.filters.StreamableFilter, weka.filters.UnsupervisedFilter)- weka.filters.unsupervised.attribute.**MergeTwoValues** (implements weka.core.OptionHandler, weka.filters.StreamableFilter, weka.filters.UnsupervisedFilter)- weka.filters.unsupervised.attribute.**MultiInstanceToPropositional** (implements weka.core.MultiInstanceCapabilitiesHandler, weka.core.OptionHandler, weka.filters.UnsupervisedFilter)- weka.filters.unsupervised.attribute.**NominalToBinary** (implements weka.core.OptionHandler, weka.filters.UnsupervisedFilter)- weka.filters.unsupervised.attribute.**NominalToString** (implements weka.core.OptionHandler, weka.filters.UnsupervisedFilter)- weka.filters.unsupervised.attribute.**NumericTransform** (implements weka.core.OptionHandler, weka.filters.StreamableFilter, weka.filters.UnsupervisedFilter)- weka.filters.unsupervised.attribute.**Obfuscate** (implements weka.filters.StreamableFilter, weka.filters.UnsupervisedFilter)- weka.filters.unsupervised.attribute.**PotentialClassIgnorer** (implements weka.core.OptionHandler)
                                          - weka.filters.unsupervised.attribute.**Center** (implements weka.filters.Sourcable, weka.filters.UnsupervisedFilter)- weka.filters.unsupervised.attribute.**Discretize** (implements weka.filters.UnsupervisedFilter, weka.core.WeightedInstancesHandler)
                                              - weka.filters.unsupervised.attribute.**PKIDiscretize** (implements weka.core.TechnicalInformationHandler)
                                              - weka.filters.unsupervised.attribute.**MathExpression** (implements weka.filters.UnsupervisedFilter)- weka.filters.unsupervised.attribute.**Normalize** (implements weka.core.OptionHandler, weka.filters.Sourcable, weka.filters.UnsupervisedFilter)- weka.filters.unsupervised.attribute.**NumericToBinary** (implements weka.filters.StreamableFilter, weka.filters.UnsupervisedFilter)- weka.filters.unsupervised.attribute.**ReplaceMissingValues** (implements weka.filters.Sourcable, weka.filters.UnsupervisedFilter)- weka.filters.unsupervised.attribute.**Standardize** (implements weka.filters.Sourcable, weka.filters.UnsupervisedFilter)
                                          - weka.filters.unsupervised.attribute.**PrincipalComponents** (implements weka.core.OptionHandler, weka.filters.UnsupervisedFilter)- weka.filters.unsupervised.attribute.**PropositionalToMultiInstance** (implements weka.core.OptionHandler, weka.filters.UnsupervisedFilter)- weka.filters.unsupervised.attribute.**RandomProjection** (implements weka.core.OptionHandler, weka.core.TechnicalInformationHandler, weka.filters.UnsupervisedFilter)- weka.filters.unsupervised.attribute.**Remove** (implements weka.core.OptionHandler, weka.filters.StreamableFilter, weka.filters.UnsupervisedFilter)- weka.filters.unsupervised.attribute.**RemoveType** (implements weka.core.OptionHandler, weka.filters.StreamableFilter, weka.filters.UnsupervisedFilter)- weka.filters.unsupervised.attribute.**RemoveUseless** (implements weka.core.OptionHandler, weka.filters.UnsupervisedFilter)- weka.filters.unsupervised.attribute.**Reorder** (implements weka.core.OptionHandler, weka.filters.StreamableFilter, weka.filters.UnsupervisedFilter)- weka.filters.**SimpleFilter** (implements weka.core.OptionHandler)
                                                          - weka.filters.**SimpleBatchFilter**
                                                            - weka.filters.unsupervised.attribute.**InterquartileRange**- weka.filters.unsupervised.attribute.**KernelFilter** (implements weka.core.TechnicalInformationHandler, weka.filters.UnsupervisedFilter)- weka.filters.unsupervised.attribute.**NumericToNominal**- weka.filters.unsupervised.attribute.**PartitionedMultiFilter**- weka.filters.unsupervised.attribute.**RELAGGS** (implements weka.core.TechnicalInformationHandler)- weka.filters.unsupervised.attribute.**Wavelet** (implements weka.core.TechnicalInformationHandler)
                                                            - weka.filters.**SimpleStreamFilter** (implements weka.filters.StreamableFilter)
                                                              - weka.filters.unsupervised.attribute.**ClassAssigner**- weka.filters.unsupervised.attribute.**NumericCleaner**- weka.filters.unsupervised.attribute.**RandomSubset**
                                                          - weka.filters.unsupervised.attribute.**StringToNominal** (implements weka.core.OptionHandler, weka.filters.UnsupervisedFilter)- weka.filters.unsupervised.attribute.**StringToWordVector** (implements weka.core.OptionHandler, weka.filters.UnsupervisedFilter)- weka.filters.unsupervised.attribute.**SwapValues** (implements weka.core.OptionHandler, weka.filters.StreamableFilter, weka.filters.UnsupervisedFilter)

---


|  |  |  |
| --- | --- | --- |
| **Overview** \| **Package** \| Class \| **Tree** \| **Deprecated** \| **Index** \| **Help** \| **Weka's home** | |  |
| **PREV**   **NEXT** | **FRAMES**    **NO FRAMES**     **All Classes** |  |


---
